# Antibiotic Discovery: Combatting Bacterial Resistance in Cells and in Biofilm Communities

**DOI:** 10.3390/molecules20045286

**Published:** 2015-03-24

**Authors:** Anahit Penesyan, Michael Gillings, Ian T. Paulsen

**Affiliations:** 1 Department of Chemistry and Biomolecular Sciences, Faculty of Science and Engineering, Macquarie University, Sydney, NSW 2109, Australia; E-Mail: ian.paulsen@mq.edu.au; 2 Department of Biological Sciences, Faculty of Science and Engineering, Macquarie University, Sydney, NSW 2109, Australia; E-Mail: michael.gillings@mq.edu.au

**Keywords:** infection control, opportunistic pathogens, bacterial evolution, eDNA, quorum sensing, biofilm matrix, unculturable microorganisms, drug discovery, natural products

## Abstract

Bacterial resistance is a rapidly escalating threat to public health as our arsenal of effective antibiotics dwindles. Therefore, there is an urgent need for new antibiotics. Drug discovery has historically focused on bacteria growing in planktonic cultures. Many antibiotics were originally developed to target individual bacterial cells, being assessed *in vitro* against microorganisms in a planktonic mode of life. However, towards the end of the 20th century it became clear that many bacteria live as complex communities called biofilms in their natural habitat, and this includes habitats within a human host. The biofilm mode of life provides advantages to microorganisms, such as enhanced resistance towards environmental stresses, including antibiotic challenge. The community level resistance provided by biofilms is distinct from resistance mechanisms that operate at a cellular level, and cannot be overlooked in the development of novel strategies to combat infectious diseases. The review compares mechanisms of antibiotic resistance at cellular and community levels in the light of past and present antibiotic discovery efforts. Future perspectives on novel strategies for treatment of biofilm-related infectious diseases are explored.

## 1. Antibiotic Resistance 

The discovery of penicillin opened a new era in the treatment of infectious diseases, described as the “golden age” of antibiotic research (1940–1962) [1]. Discovery of other antimicrobials soon followed, and included widely used antibiotics including streptomycin, chloramphenicol, and tetracycline. For the first time, many common bacterial diseases could be cured. Moreover, the first antibiotics played a crucial role in the treatment and prevention of infections during World War II [2]. Antibiotics were so successful that they were considered the ultimate cure, the “miracle drugs” which the medical world was craving. As a result of the initial success of antibiotics, bacterial diseases were naively considered to be permanently defeated.

However, with increasing use of antibiotics, more and more pathogenic bacteria developed resistance to their inhibitory effects [3]. Consequently, despite their initial effectiveness, most antibiotics have a limited life, and from their first introduction they select for pathogen variants that have intrinsic or acquired resistance mechanisms [4]. Currently, antimicrobial resistance threatens the effective prevention and treatment of an ever-expanding range of infections. It is an increasingly serious threat to global public health that requires immediate action, and affects all parts of the world as new resistance mechanisms emerge and rapidly spread around the globe [5].

**Figure 1:**
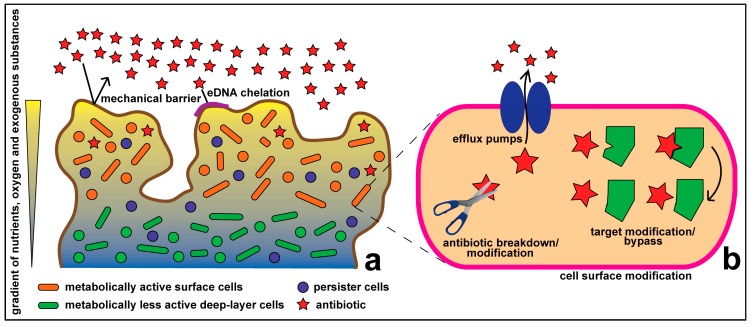
Antibiotic resistance at the community (**a**) and cellular (**b**) levels.

In recent years, we have gained a better understanding of the intra- and inter-cellular processes that govern bacterial ecology. Far from being isolated cells, at least some bacteria are perhaps more appropriately viewed as disseminated multicellular organisms, whose interactions are mediated by complex cell-cell signaling [6,7]. Cell-cell interactions can lead to the formation of spatially complex matrices of polysaccharide and extracellular DNA into which cells are embedded to form a biofilm community [8]. Combatting bacterial infections thus requires both an understanding of intracellular genetics and biochemistry, and an understanding of how the biofilm mode of life affects antibiotic uptake and resistance (Figure 1).

### 1.1. Resistance at the Cellular Level

Traditional understanding of antibiotic resistance deals with how resistance occurs within an individual microbial cell. Historically, the majority of research on antibiotic resistance has been focused on cellular resistance, which includes such classical mechanisms as: inactivation of drugs via hydrolysis (e.g., via β-lactamase) or modification (e.g., aminoglycoside resistance); alteration of drug targets within cells thus making them unrecognizable to the drug (e.g., by mutating DNA gyrase in fluoroquinolone resistance) or bypassing the drug target; the use of permeation barriers, preventing access of drugs to the target (e.g., the Gram-negative outer membrane); and active efflux of drugs out of the cell via membrane-bound efflux transporters [9,10] (Figure 1). 

The development of cellular resistance occurs as a result of mutations to endogenous genes, and via lateral gene transfer of resistance determinants from other microorganisms. Recent advances in genomics and metagenomics have revealed that many natural ecosystems, including diverse environments such as the human gut and soil, contain large number of genes whose functions can be co-opted to confer resistance to antimicrobials [11,12,13,14]. These genes are collectively known as the resistome [13,15,16]. 

The resistome concept is anthropocentric, since the original functions of the genes that comprise the resistome were probably not to confer antibiotic resistance phenotypes. However, the resistome concept is certainly useful, since it underscores the role of environmental bacteria in supplying resistance genes to pathogens [17]. The recovery of genes that can confer resistance phenotypes from extreme environments that have not been in contact with humans, such as the deep subsurface [18], ice [19] and permafrost [20], further suggests that these genes have natural roles other than conferring antibiotic resistance. Resistance mechanisms such as multridrug transporters might have evolved as transporters for naturally occurring substrates, serving as mechanisms to pump toxins from cells, and their ability to also transport antibiotics may be fortuitous [21]. “Resistance” genes during the pre-antibiotic period were probably chromosomal, and encoded functions of physiological importance. In the post antibiotic period, resistome genes were laterally transferred to a new host where they lacked their original biochemical and genetic context, and their functions became limited to antibiotic resistance [22].

Over the last fifty years research into resistance has mainly focused on clinical aspects of antibiotic resistance, while the possible original functions of resistance genes have been largely overlooked. Understanding the original roles of these resistome elements may aid the development of successful strategies to fight infections caused by antibiotic resistant pathogens.

### 1.2. Community Level Resistance 

Bacterial communities can exhibit tolerance to environmental stress that single cells cannot, and this we refer to as community level resistance. Such tolerance can extend to include an increased resistance to antibiotics. For instance, microbes in a biofilm community gain additional antibiotic resistance that can be up to 1000 times higher than the corresponding planktonic cells [23]. Community level resistance adds to the cellular level resistance, thus greatly enhancing the overall antibiotic resistance of the microbial community (Figure 1).

In their natural habitats, microorganisms predominantly live in communities: biofilms composed of tightly packed cell aggregates encased within a secreted matrix that includes exopolysaccharides, amyloid fibers and extracellular DNA (eDNA) [8,24,25]. These aggregates are characterized by the presence of strong nutrient and oxygen gradients that may lead to heterogeneity and bacterial cell differentiation. Cells in the deeper layers of biofilms may have a slower metabolism, being locally adapted to the nutrient and oxygen limited conditions compared to the more metabolically active surface cells. This, in turn, can lead to significant differences in resistance exhibited by these subpopulations in response to antimicrobials [26].

It is generally accepted that the majority of bacteria live in biofilms, both in natural environments such as soil and water, and within the human host [27]. Despite this observation, research on antibiotic resistance has historically focused on planktonic cultures, and thus the contribution of community resistance has been largely ignored. Even in the pharmaceutical industry, levels of drug resistance are often assessed on planktonic cultures. When 80% of all infections are complicated by involvement of biofilms [28], guidelines for antibiotic use based on planktonic cells may be ineffective due to the added community level resistance of biofilms.

Biofilm-specific resistance mechanisms, which are distinct from the well-characterized cellular level resistance mechanisms, may act in an orchestrated manner to confer high levels of antibiotic resistance in biofilm communities (Figure 1). Components of the biofilm matrix form a mechanical shield and also act to inhibit the effect of antibiotics. The Pel and Psl polysaccharides, produced in *Pseudomonas aeruginosa* biofilms, contribute to antibiotic resistance. Pel deficient mutants are more susceptible to aminoglycoside antibiotics tobramycin and gentamicin compared to the wild type [29,30].

Extracellular DNA (eDNA) forms part of biofilm matrices [25], and may have a role in biofilm antibiotic resistance. Because eDNA is negatively charged, it can act as a chelator of cationic antimicrobials [31] and has been shown to be involved in resistance towards cationic peptides [31]. Extracellular DNA can also act as a shield against aminoglycosides [32].

Bacteria can become highly resistant to antibiotics when they experience nutrient limitation in growth media [33]. This probably also applies to cells in biofilms because cells in deep layers of the biofilm may experience nutrient limitation, leading to a similar increase in resistance [26,34]. The starvation-induced stringent (SOS) response has been implicated in enhanced biofilm-specific resistance towards various classes of antibiotics in organisms such as *P. aeruginosa* and *Escherichia coli* [33,35].

Another phenomenon that greatly contributes to antibiotic resistance in biofilms is the emergence of persister cells [36] that are more prevalent in biofilms compared to planktonic cultures. Persister cells adopt a slow or non-growing phenotype and are highly resistant to environmental stresses, including antibiotic challenge [34]. Thus, many antibiotics, for example, β-lactams, that target growth-specific factors and are active against dividing bacterial cells, will have a limited effect against this cell population. Furthermore, persister cells may survive antibiotic treatments even when the rest of the community has perished, thus creating reservoirs of surviving cells that are able to regrow and cause relapsing infections [37]. Metabolic quiescence is a strategy for tolerating antibiotic exposure, as demonstrated by lag time mutants, which survive high-level antibiotic exposure, and are the first adaptive changes to be seen in some experimental situations [38]. 

### 1.3. Synergy between Community and Cellular Level Resistance Mechanisms 

Despite the inherent differences in the nature and mechanisms of cellular and community resistance, they are synergistic. The biofilm mode of life, besides providing community level resistance, can also promote cellular level resistance. Biofilms have a greatly enhanced mutation rate (up to 100 times higher than planktonic cells) [39] which inevitably leads to faster development of antibiotic resistant mutants. Moreover, the close proximity of various microbial organisms within biofilm aggregates and the abundance of eDNA likely facilitate horizontal gene transfer and acquisition and spread of resistance determinants. Indeed, it has been shown that biofilms may constitute specific foci of genetic adaptation and evolution, leading to the selection of subpopulations with a greater ability to acquire antibiotic resistance [40,41] and the horizontal acquisition of exogenous DNA [42,43]. 

Biofilms promote the acquisition and exchange of integron gene cassettes [44,45], many of which encode antibiotic resistance. Biofilms in animal digestive systems, aquatic environments, the rhizosphere and phyllosphere also promote conjugation and natural transformation [46,47,48,49]. Basal rates of bacterial evolution are thus accelerated in biofilms, especially when exposed to sub-inhibitory concentrations of antibiotics [50]. Because the barrier effect of the biofilm matrix can significantly decrease the penetration of drugs, the resulting sub-inhibitory concentration of antibiotics in parts of the biofilm creates favorable conditions for selection of resistant phenotypes, without the cells being exposed to lethal levels of the antibiotic. Furthermore, exposure to sub-inhibitory antibiotic concentrations induces increased rates of mutation, recombination and lateral transfer [50,51].

In addition, traditional mechanisms of cellular level resistance can also act in a biofilm-specific manner. For example, an up-regulation of certain drug efflux pumps is observed in *P. aeruginosa* [52] and *E. coli* [53] biofilms even without an antibiotic challenge, suggesting their possible role in the biofilm mode of life.

## 2. Antibiotic Discovery

### 2.1. The Past and the Present

Antibiotics are defined as compounds that can effectively inhibit the growth of microorganisms. They have been used for the treatment of bacterial diseases since the early 20th century. After the introduction of penicillin, many classes of antibiotics were discovered and most infectious diseases were brought under control. However, the increased use of antibiotics in clinical practice was soon followed by the emergence of antibiotic resistance. Indeed, resistance started appearing in target organisms within a few years of introduction of antibiotics into medical practice [54]. As an example, within seven years of penicillin’s first use, 50% of hospital *Staphylococcus aureus* isolates were resistant [55].

The possibility of finding an ultimate cure for bacterial disease proved to be an illusion. As of 2004, more than 70% of pathogenic bacteria were resistant to at least one of the currently used antibiotics [56]. Humanity is involved in a continuous struggle against bacterial resistance, requiring the constant development and supply of novel antimicrobials to tackle ever more resistant pathogens [57,58,59].

Chemical syntheses and high-throughput screening of chemical libraries against defined macromolecular targets are some of the more recent approaches of antibiotic discovery. However, the first libraries of chemically synthesized compounds provided more quantity than quality. For example, GlaxoSmithKline recently disclosed the results of a campaign to discover broad-spectrum antibiotics. After seven years of research the campaign was abandoned because of the limited chemical diversity of their synthetic screening libraries [60].

Many currently used antibiotics are derived from natural products, as they provide diversity and structural complexity with densely packed functional groups; properties that make chemical synthesis of these compounds extremely difficult [1,61,62]. Natural systems provide a great source of biologically active compounds. In addition to traditional terrestrial environments, in recent years underexplored habitats, such as marine and hypersaline environments, have increasingly been targeted as new sources for the discovery of organisms producing novel antimicrobial molecules [59,63,64,65]. In 2012, over 1200 novel natural products were discovered from marine sources alone [66], an 8% increase in the number of compounds reported in 2011 [67]. Overall, the number of currently known natural products exceeds 1 million compounds [68]. 

Advances in molecular biological techniques, including metagenomics and functional screening, have provided an additional avenue for the discovery of new compounds. Such techniques allow access to unculturable organisms via screening gene products obtained by expressing genes recovered directly from the environment. This bypasses the need for culturing the original organism. Since 99% of microorganisms are currently considered to be unculturable, this approach significantly deepens the pool for source organisms [69,70].

Advanced culturing methods can also assist in finding novel antibiotics from previously uncultured microorganisms. Teixobactin, the first member of a new class of lipid II binding antibiotics, was obtained from a previously uncultured bacterium using a specially developed multichannel device for isolating and growing microorganisms *in situ* within their natural soil environment [71]. The vast untapped resource of yet to be cultured organisms may be the next source of novel antimicrobial compounds.

Combinatorial biosynthesis and synthetic biology techniques that express genes from different biosynthetic pathways can generate libraries of hybrid structures. However, in practice, this approach is problematic. Firstly, it involves the construction of various recombinant organisms which is labor-intensive and costly. Secondly, hybrid biosynthetic pathways rely on enzymes having low substrate specificity, which is not always the case [72,73]. Nevertheless, despite challenges, there has been substantial progress in this area over the past decades [74,75,76].

Unfortunately, despite the potential of these approaches and the desperate need for new antibiotics, there has been little investment into antibiotic discovery by the pharmaceutical industry, largely because financial returns are likely to be limited. Development of antibiotics faces stringent government regulations that can delay new drugs entering the market [77]. The time between initial discovery of a compound and entering the market takes 10 years on average. This means that antibiotics launched today are the products of drug discovery projects initiated a decade ago [78]. 

Due to the expense involved in developing new antibiotics and the low probability of recovering the costs once the antibiotic is marketed, the pharmaceutical industry frequently prefers to invest in drugs for chronic diseases and lifestyle drugs that provide a long-term revenue stream [55,79]. If the current trends continue, we may soon return to a situation where there is no effective cure for resistant pathogens. Recent global efforts, including statements by the WHO and CDC, drawing attention to bacterial resistance and the urgency of tackling this problem, may help to avoid this finale.

### 2.2. Future Perspectives 

Traditionally the focus of antibiotic discovery has been on discovering compounds that target cellular mechanisms in the planktonic mode of growth, both *in vitro* and *in vivo*. As a result, many antibiotics are less effective against microbes in biofilms. This is of particular concern, given that we now know that biofilms play a role in many infections. Research on biofilms is an expanding area, as the first biofilms were only described towards the end of 20th century [27]. Poor understanding of the biofilm mode of life has retarded the development of drugs that specifically target biofilms [26]. In recent years, with increased failure in the treatment of infectious diseases, there has been a shift toward realization of the importance of developing anti-biofilm drugs and several strategies have been explored.

As part of the natural biofilm development cycle, cells within mature biofilms produce compounds that can induce their shift from biofilm to a planktonic mode of life. This shift is essential in the process of dispersal from biofilms [80]. Dispersal processes confer a significant ecological advantage as it allows the dissemination of bacterial populations to colonize new habitats. This property of biofilms has been exploited in the development of anti-biofilm drugs via identification and characterization of such chemical cues. For example, exogenous addition of d-amino acids, which are naturally produced by dispersing cells of the Gram-positive bacterium *Bacillus subtilis*, led to the dispersal of *B. subtilis* biofilms [81]. This approach can also inhibit biofilm formation by other organisms, including the Gram-positive pathogen *S. aureus* and the Gram-negative pathogen *P. aeruginosa* [82]. d-amino acids were reportedly involved in the release of amyloid fibers—part of the matrix that links cells within biofilms [81]. Similarly, the self-produced polyamine norspermidine that targets exopolysaccharides within the biofilm matrix was reported to lead to the disruption of *B. subtilis* biofilms and prevent biofilm formation by *S. aureus* and *E. coli* [81]. 

Nitric oxide (NO), a signaling molecule found in many organisms, has also been implicated in biofilm dispersal. Thus, the exogenous addition of non-toxic levels of NO was shown to stimulate phosphodiesterases that degrade c-di-GMP, an essential regulator of biofilm formation and dispersal, thus triggering a switch to a planktonic phenotype [83,84]. 

Another strategy to target biofilms is the use of synthetic cationic peptides derived from various natural products [26]. For example, it has been noted that the natural human peptide LL-37 was able to both prevent and disperse biofilms [85]. This prompted further development of an improved and smaller synthetic peptide with anti-biofilm properties, based on LL-37 [86]. 

An alternative strategy to prevent biofilm formation is via targeting cell signaling, such as quorum sensing, that is necessary for cells to form and maintain biofilms. Quorum sensing is a population density-dependent signaling system that acts via production of often diffusible signaling molecules, such as the acylated homoserine lactones (AHLs) of Gram-negative bacteria. In this process, signaling molecules reach a threshold concentration in the environment, driven by the number of producer cells in the local environment. This triggers community responses, including biofilm formation and production of virulence factors. The use of molecules that have structural similarity to quorum sensing signals is another potential approach to prevent biofilm formation. For example, algal-derived furanones and their synthetic analogs with structural similarity to AHLs, are able to reduce quorum sensing effects by presumably blocking AHL binding sites; this has proven a viable strategy against biofilm formation [87,88,89].

It should be noted that drugs that prevent biofilm formation or lead to their dispersal, have an inherent disadvantage if they do not affect growth of individual cells. They need to be continuously applied as the removal of the drug can potentially lead to rapid re-establishment of biofilms by existing planktonic cells. Therefore, a combination therapy, applying anti-biofilm drugs in conjunction with traditional antibiotics that target cell growth, could be a better alternative in the control of biofilm-related infectious diseases. In such combination therapy, the anti-biofilm drugs will promote planktonic growth, thus removing the additional community level resistance provided by biofilms, and facilitate the targeting of pathogens at the cellular level by traditional antibiotics.
